# Metabolic Effects of Social Isolation in Adult C57BL/6 Mice

**DOI:** 10.1155/2014/690950

**Published:** 2014-11-26

**Authors:** Meng Sun, Eugene Y. Choi, Daniel J. Magee, Colin W. Stets, Matthew J. During, En-Ju D. Lin

**Affiliations:** ^1^Department of Molecular Virology, Immunology and Medical Genetics and the Comprehensive Cancer Center, The Ohio State University, 912 Biomedical Research Tower, 460 West 12th Avenue, Columbus, OH 43210, USA; ^2^Functional Genomics and Translational Neuroscience Lab, Department of Molecular Medicine and Pathology, University of Auckland, Auckland 1142, New Zealand

## Abstract

Obesity and metabolic dysfunction are risk factors for a number of chronic diseases, such as type 2 diabetes, hypertension, heart disease, stroke, and certain forms of cancers. Both animal studies and human population-based and clinical studies have suggested that chronic stress is a risk factor for metabolic disorders. A good social support system is known to exert positive effects on the mental and physical well-being of an individual. On the other hand, long-term deprivation of social contacts may represent a stressful condition that has negative effects on health. In the present study, we investigated the effects of chronic social isolation on metabolic parameters in adult C57BL/6 mice. We found that individually housed mice had increased adipose mass compared to group-housed mice, despite comparable body weight. The mechanism for the expansion of white adipose tissue mass was depot-specific. Notably, food intake was reduced in the social isolated animals, which occurred around the light-dark phase transition periods. Similarly, reductions in heat generated and the respiratory exchange ratio were observed during the light-dark transitions. These phase-specific changes due to long-term social isolation have not been reported previously. Our study shows social isolation contributes to increased adiposity and altered metabolic functions.

## 1. Introduction

Social isolation (SI) has been shown to induce an increase in emotionality and hypothalamic-pituitary-adrenal axis reactivity in mice [1, 2]. Long-term individual housing has been used as a chronic stress model in the study of psychiatric disorders [3–5] and, to a lesser extent, metabolic disorders [6]. Given the association between stress and obesity [7, 8], efforts have been made in developing and characterizing animal stress models for the study of metabolic dysfunctions. These models include unpredictable chronic stress model, social defeat model, and the use of various chronic stressors [9–11].

The hypothalamo-pituitary-adrenal (HPA) axis and the sympathetic nervous system (SNS) are the main effector pathways of the stress system and both affects energy metabolism. During acute stress, activation of the SNS triggers the release of epinephrine, which mediates a number of physiological changes to prepare the body for “fight or flight” response. These changes include increased heart rate, increased blood flow to vital organs, and mobilization of energy source by enhanced lipolysis and hepatic gluconeogenesis [12]. At the same time, corticotropin-releasing hormone (CRH) is released from the paraventricular nucleus (PVN) of the hypothalamus. CRH causes an immediate suppression of food intake through inhibiting the appetite-stimulating neuropeptide Y (NPY)/agouti-related peptide (AGRP) neurons in the arcuate nucleus (ARC), thereby diverting the body's resources to deal with the stressor [13–16]. CRH also induces the release of adrenocorticotropic hormone (ACTH) from the pituitary. The circulating ACTH in turn stimulates the adrenal cortex to secrete glucocorticoids, which regulate metabolism and food intake through a number of mechanisms [17, 18]. In addition to stimulating feeding responses by inhibiting CRH release and increasing NPY release in the hypothalamus [19], cortisol also upregulates genes encoding lipoprotein receptors and enzymes of glucose, lipid, and amino acid metabolism (see [18]). Moreover, stress-induced elevations in glucocorticoids secretion appear to intensify emotions and motivation [20, 21] and promote the intake of palatable foods [22, 23], thus contributing to the development of “comfort eating” [24, 25]. Ethologically, the appetite-stimulatory effect of the glucocorticoids serves to replenish the energy used when the stressful event subsides.

In a normal stress response, the activation of both the HPA axis and the SNS is of limited duration and would return to baseline when homeostasis is achieved. However, in chronic stress, prolonged activation of the stress system can lead to a disruption of homeostasis. Based on the current understanding of the stress response, it is reasonable to postulate that chronic stress lead to chronic hypercortisolemia, which promotes feeding and hence excessive weight gain. However, both human and animal studies showed that chronic stress could lead to either increase or decrease of food intake and body weight [26–30]. The reason for the bidirectional response to stress is not fully understood and is likely to involve many factors [31, 32]. One explanation is the balance between an increase in *β*-adrenergic activation, the body's main fat-burning mechanism (leading to weight loss), and the increased intake of sugar- and fat-rich comfort foods (resulting in weight gain) [25, 33, 34]. The observation that those who are initially overweight are more inclined to increase body weight when stressed whereas those who are of normal- or underweight do not led Dallman to propose that difference in metabolic outcomes might be the results of higher insulin concentration in people with higher body mass index [24]. More recently, others have suggested that individual differences in ghrelin level and response may contribute to the variability in stress-induced appetite [35].

Animal models are useful for understanding the complex interaction between stress and metabolic processes. Similar to human observation, these animal models have produced variable and even opposite phenotypes of food intake, body weight gain, and adiposity. The variable metabolic phenotypes observed in the different studies have been attributed to the types of stressors, diets, protocol durations, strains of animals, and stress intensities. The variability highlights the importance of a thorough characterization of different stress models. SI is a simple model with minimal handling and technical requirement. However, only a handful of studies directly investigated the effect of SI on body weight regulation and metabolic functions. Conflicting results were reported for the effects of SI, whereas some observed an increase [36, 37], others observed a decrease in body weight gain [38–42]. In this study, we assessed the effect of SI on body weight gain and adiposity in adult C57BL/6J mice after two months of single housing. We also monitored the food intake, activity, heat production, and respiratory exchange ratio (RER) of the mice using a metabolic chamber.

## 2. Materials and Methods

### 2.1. Animals

Twelve-week-old male C57BL/6 mice (Charles River Laboratories, Wilmington, MA) were housed in groups of four (control; *n* = 20) or single-housed (SI; *n* = 16) in standard cage (31 cm × 17 cm × 14 cm) for 8 weeks. All mice were kept under a 12 h light/dark cycle (lights on at 0600 hr), with free access to water and food (Diet 7012, Harlan Laboratories). All use of animals was approved by the Ohio State University Animal Care and Use Committee and was in accordance with the NIH guidelines.

### 2.2. Metabolic Chamber Analysis

Following eight weeks of SI (or control housing), a subset of mice (*n* = 4 per group) was individually placed in the chambers of the Oxymax Lab Animal Monitoring system (Columbus Instruments, OH). Following 2 days of acclimatization, food intake, activity, heat generated, and respiratory exchange ratio (RER) were recorded in day 3.

### 2.3. Tissue Collection

Eight weeks after the start of isolation, mice were culled by decapitation under isoflurane anesthesia. Trunk blood was collected and serum was isolated by centrifugation and stored at −20°C until assayed. Brown adipose tissue (BAT), inguinal (WATi), retroperitoneal (WATr), and epididymal (WATe) white adipose tissue were dissected and weighed to determine body composition and adiposity. WAT depots were collected from one side only, immersed in 4% paraformaldehyde and paraffin-embedded for subsequent morphological analysis. For tissue weight measurements, the subset of mice that underwent metabolic chamber analysis was not included as mice were euthanized by a later time point. One SI mouse was excluded because of a large mass found in the abdominal cavity. Tissue were dissected and weighed from 14 control mice instead of 16 due to time constraint.

### 2.4. WAT Staining and Adipocyte Size Analysis

Paraffin-embedded WAT was cut into 20 *μ*m sections using a cryostat and mounted on slides. Sections were stained with hematoxylin and eosin (H&E) to examine the degree of adipocyte hypertrophy. Adipocyte diameter was measured using the Quick Measure Circle Command in Stereo Investigator 7 (MBF Bioscience, Willeston, VT) using a 10x objective. Software generated 150 *μ*m grids were drawn over the entire section; the cells within every other grid were circled to estimate diameter of the circle, excluding any incomplete cells. At least 10 cells per section were measured and then averaged.

### 2.5. Serum Biomarker Analysis by ELISA

Serum was analyzed for leptin, leptin receptor, adiponectin, and insulin-like growth factor-1 (IGF-1) using DuoSet ELISA Development Systems (R&D Systems, Minneapolis, MN) according to manufacturer's instruction. Serum corticosterone level was determined using Enzyme Immunoassay Kit at 1 : 200 dilution according to the manufacturer's instruction (Assay Designs, Inc., Ann Arbor, MI).

### 2.6. Statistical Analysis

Statistical analysis was performed using JMP software (SAS Institute Inc., Cary, NC). For food intake, activity, heat generated, and RER over time, we determined the overall significance by repeated measure ANOVA while pair-wise comparison of individual time-points was assessed using Student's *t*-test. All other results were analyzed by Student's *t*-test for significant differences between the control and the isolation groups. Statistical significance was set at *P* < 0.05. All data are presented as means ± standard error of the mean (S.E.M).

## 3. Results

### 3.1. SI Increased Adiposity without Increase in Body Weight

**Figure 1:**
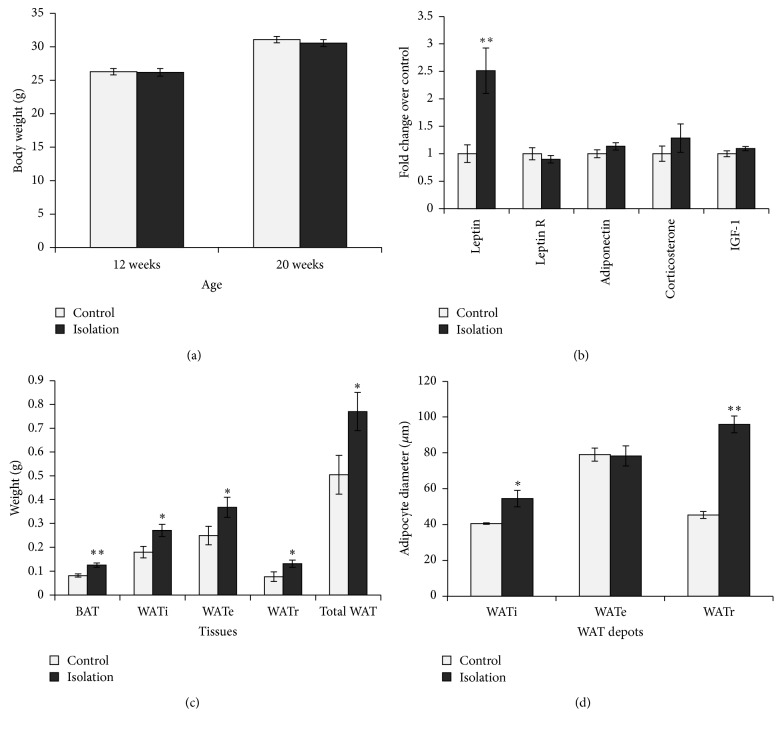
(a) Body weight at the start of the experiment (12 weeks) and following 2 months of differential housing conditions at 20 weeks was comparable between the groups (*n* = 11 for SI group, *n* = 14 for group-housed control). (b) Serum biomarker profile after 2 months of social isolation showed robust increase in leptin level (*n* = 7–20 per group). (c) Interscapular BAT and WAT depots (from one side) were all increased by SI (SI, *n* = 11, control, *n* = 14). (d) Adipocyte diameters were greater in WATi and WATr but not WATe in the SI mice (*n* = 3–5 mice per group, each mouse with over 10 measurements). Data shown are mean ± S.E.M. ^*^ *P* < 0.05. ^**^ *P* < 0.01.

Eight weeks of social isolation significantly increased adiposity without significant body weight gain (Figures 1(a) and 1(c)). Brown adipose tissue (BAT) weight was increased by 1.54-fold (*P* < 0.01). WATi, WATe, and WATr weights showed increases of 1.51-fold (*P* < 0.05), 1.48-fold (*P* < 0.05), and 1.72-fold (*P* < 0.05), respectively. The combined WAT from the three depots was increased by 1.53-fold (*P* < 0.05) in the single-housed mice. Consistent with the increase in adiposity, the SI group showed marked increase in serum leptin level (*P* < 0.01, Figure 1(b)). In contrast, levels of serum adiponectin, corticosterone, IGF-1, and leptin receptor were comparable between isolated and group-housed mice.

### 3.2. SI Increased WAT Tissue Mass via Distinct Mechanisms in Different Depots

**Figure 2:**
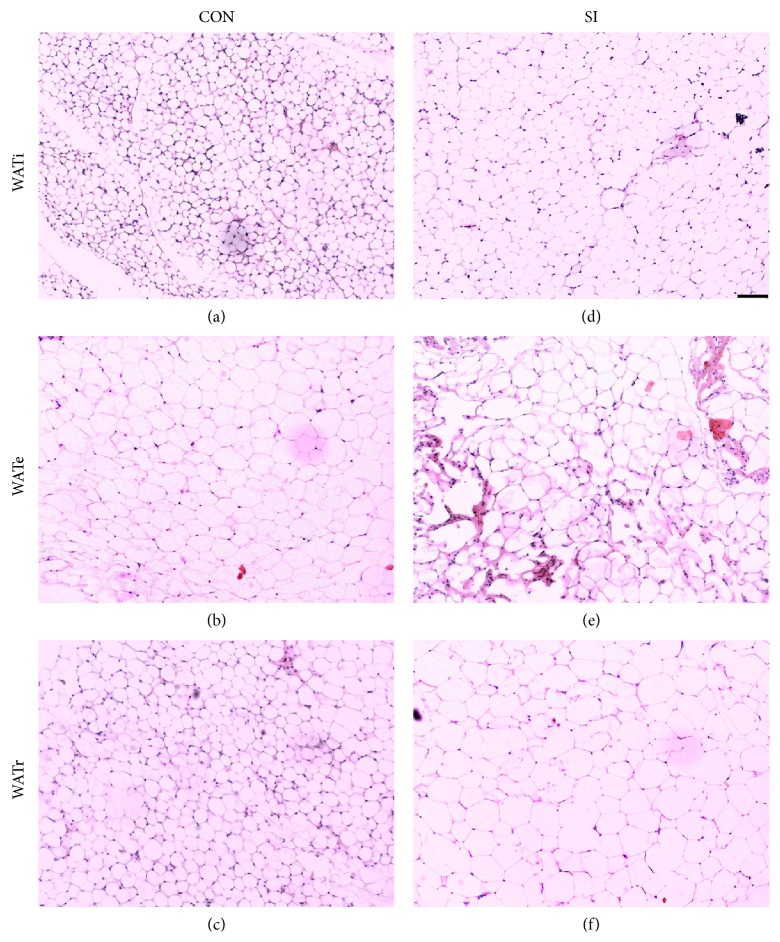
Representative sections of H&E stained adipose tissue showing WATi and WATr adipocytes were enlarged in SI mice ((d) and (f), resp.) compared to control C57BL/6 mice ((a) and (c), resp.). In contrast, WATe adipocytes were comparable in size between the two groups ((b) and (e)). Scale bar = 100 *μ*m.

We compared the adipocyte diameters in the two groups to examine whether the increase in WAT tissue mass was due to increased adipocyte number (hyperplasia) or increase in adipocyte size (hypertrophy). Interestingly, the effects were depot-specific. In the SI group, the adipocytes were significantly larger for WATi and WATr, by 1.3- (*P* < 0.05) and 2.1- (*P* < 0.01) fold, respectively. In contrast, the cell sizes of WATe were comparable between the two groups (Figure 1(d)).  Figure 2 shows representative sections of the different WAT depots in the control and isolated mice.

### 3.3. SI Reduced Food Intake and RER at the Light-Dark Transition Periods

**Figure 3:**
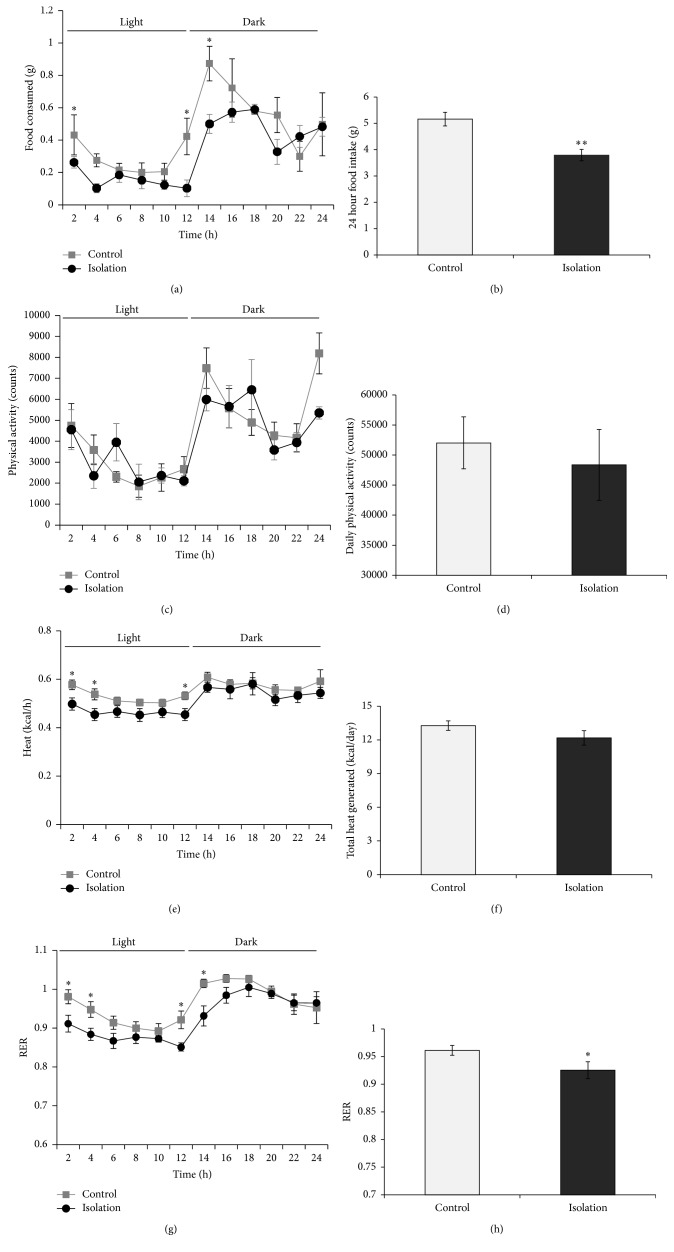
Effects of SI on food intake ((a) and (b)), physical activity ((c) and (d)), energy expenditure ((e) and (f)), and respiratory exchange ratio ((g) and (h)) (*n* = 4 per group). Social isolation caused a reduction in food intake (a) and RER (g) and a subtle effect on energy expenditure (heat (e)) around light-dark transition periods. Data shown are mean ± S.E.M. ^*^ *P* < 0.05. ^**^ *P* < 0.01.

Daily food intake was reduced in the SI group (*P* < 0.01, Figure 3(b)). Repeated measure ANOVA revealed a significant effect over time (*P* < 0.0001), although the interaction effect between time and housing condition was not significant (*P* = 0.202). A group effect was detected (*P* < 0.01). Notably, the difference in food intake between the two groups was most dramatic at the transition periods between light and dark phases (Figure 3(a)).

A similar observation was made for energy expenditure and RER over time (Figures 3(e) and 3(g)), with differences detected at the transition periods between light and dark phases. Interaction effects were detected between time and housing condition for both energy expenditure (*P* < 0.05) and RER (*P* < 0.05), indicating that SI exerted differential effects depending on the time of day. The reduction in energy expenditure in the isolated mice was subtle and did not reach statistical significance when total daily energy expenditure was compared (Figure 3(f)). In contrast, the RER was significantly lower in the SI group (*P* < 0.05; Figure 3(h)), suggesting a shift from carbohydrate to lipid oxidation. Daily physical activity was unaffected by SI (Figures 3(c) and 3(d)).

## 4. Discussion

Animal models of chronic stress are important tools for the study of the pathophysiology and molecular mediators of stress-related disorders such as anxiety and metabolic syndromes. These animal models are also needed for the verification of potential treatments. Our study shows that two months of SI can produce profound effect on body composition despite minimal effect on body weight. In line with this observation, a recent report also showed that 4-week-old C56BL/6J mice individually housed for 13 weeks had increased body weight and visceral fat compared to group-housed mice, evident after 9 weeks of SI [37]. Here we observed an increase in adiposity prior to any significant body weight gain. Although it remains possible that longer duration of social isolation may eventually lead to significant increases in body weight gain, our study shows that there was a change in body composition and a preferential accumulation of adipose tissues even before any observable body weight gain.

Consistent with the increased adipose mass, the SI mice exhibited a marked increase in serum leptin. Interestingly, we did not observe changes in serum adiponectin, which often change in opposite direction to serum leptin and are known to decrease under obese conditions [43–45]. A previous study by Sakakibara et al. found decreased plasma adiponectin after 13 weeks of social isolation, correlating to an increased body weight gain of the isolated mice, but intriguingly plasma leptin was unchanged [37]. In contrast, our finding is similar to that reported by Nonogaki et al., in which the socially isolated mice exhibited increased WATe weight, BAT weight, and plasma leptin, but no change in plasma adiponectin level [6]. In human, obesity can lead to marked changes in the growth hormone (GH)—IGF-1 axis [46]. Both increases and decreases in circulating IGF-1 have been reported in obese subjects [47–50]. Chronic stress can also lead to decreased IGF-1 levels [51, 52]. In the present study, we did not observe any significant change in serum IGF-1 level. This may reflect the lack of body weight change in the SI mice and indicates that chronic social isolation stress exerts a greater effect on adiposity with little impact on the somatotropic axis.

Serum corticosterone was unchanged in the SI mice after 8 weeks of individual housing, consistent with a recent study that showed comparable basal corticosterone level in adult C57BL/6J male mice after 4 weeks of social isolation [1]. This was likely due to an adaptation to the chronic stress. A return to comparable baseline corticosterone following chronic psychosocial stress has been reported by the others using the chronic subordinate colony housing paradigm in mice [53, 54]. Similarly, using a social overcrowding stress model, we observed an increase in serum corticosterone in the first three weeks and then returned to baseline by 8 weeks [55]. It is interesting to note that although baseline corticosterone might have returned to normal, these mice could exhibit enhanced stress-induced corticosterone release as shown in a previous study [1].

Although all WAT depots were enlarged, only WATi and WATr showed an increase in adipocyte size, indicating that the enlargement of these depots were at least in part due to adipocyte hypertrophy. In contrast, WATe adipocyte sizes were comparable between the SI and control mice, suggesting that the enlargement of this particular fat depot was due to adipose hyperplasia rather than hypertrophy. Thus, the SI model may provide us a way to study the different mechanisms of adipose tissue expansion. The hypertrophy of adipocytes can also trigger a chronic low-grade inflammatory state as adipocytes become distressed and undergo necrosis/apoptosis, consequently recruiting macrophages [18, 56]. In addition, as fat accumulates, adipokines and chemokines are secreted from adipocytes into the systemic circulation, attracting macrophages from the bloodstream into the expanding adipose tissue. In turn these resident macrophages release more cytokines (e.g., TNF-*α* and IL-6), which further stimulates the secretion of proinflammatory adipokines, resulting in a vicious cycle between adipocytes and macrophages inside the fat depot [57]. Given the different mechanisms of tissue expansion of the different WAT depots, it may be interesting to study the local inflammatory and immune response (cytokines) of the various WAT depots.

Although the present study focused on the WAT due to their direct relevance in obesity, the mechanism and effects of the enlargement of the BAT in stress-induced metabolic dysfunction warrant further study. BAT is the main site of nonshivering thermogenesis in mammals that dissipate energy through heat production. Three weeks of isolation was shown to increase both uncoupling protein-1 (UCP-1) content and sympathetic outflow in the BAT in rats [58]. Indeed, enhanced activation of BAT due to psychological stress has long been noted [59]. However, the role of this psychological stress-induced hyperthermia or “psychogenic fever” in stress-related metabolic changes is unclear.

In contrast to the increased food intake observed by Sakakibara et al. [37], we found a reduction in food intake in the SI animals. A couple of notable differences between the two studies may contribute to this discrepancy. First, they used 4 week old mice instead of 12 week old adult mice. Secondly, they used reduced bedding to induce a more severe stress response. Taken together, our data suggest that increase in food intake was unlikely a primary cause for the increase in adiposity caused by isolation stress. The same group also reported changes in a lipid metabolism-related pathway in the liver of SI mice prior to body weight change [60]. Gene expression changes favoring the activation of the lipid biosynthesis pathway controlled by sterol regulatory element binding factor 1 (srebf1) would likely lead to the accumulation of fat. Another study reported an upregulation of metabolic genes in the mammary adipocytes of SI mice that coincided with increased lipid synthesis and leptin secretion [61]. Thus, changes in adipocyte gene expression and physiology are likely to result in the increase in adiposity. It is also possible that the increase in adiposity without body weight change was a result of differential energy partitioning, since SI can lead to reductions in bone mineral content and soft-lean tissue mass [38].

The metabolic chamber monitoring also revealed decreases in RER and heat produced in the SI mice, around times of light-dark transitions. On the other hand, activity level was comparable between single- and group-housed mice, although others have reported increased locomotion in socially isolated mice [62]. One factor to consider is the potentially greater stress in the group-housed mice when subjected to individual housing in the metabolic chamber, which may affect activity level. The reduced RER indicates that SI mice utilized more fatty acid as energy source compared to group-housed mice, which used more carbohydrate. This is consistent with the higher adiposity of the SI mice. Although the total heat produced was not significantly different between the two groups, the reduction in heat production during certain times of the day in the SI mice might contribute to a lower energy expenditure over time, contributing to an increase in adiposity in the SI mice.

## 5. Conclusions

In summary, here we show that long-term social isolation induced an increase in adiposity prior to any significant body weight gain. The expansion of adipose tissue was due to hyperplasia in the WATe and hypertrophy (with or without hyperplasia) in the WATi and WATr. These animals also exhibited blunted metabolic responses during light-dark transition that warrants further study.
